# Development of a Multiplex PCR Assay for Simultaneous Identification of Six Commercially Important Bivalves

**DOI:** 10.3390/foods14223881

**Published:** 2025-11-13

**Authors:** Kang-Rae Kim, Hye-Jin Kim, In-Chul Bang

**Affiliations:** 1 Southeast Sea Fisheries Research Institute, National Institute of Fisheries Science, Namhae 52440, Republic of Korea; kimkangrae9586@gmail.com; 2 Department of Life Science, Soonchunhyang University, Asan 336-745, Republic of Korea; n_siho@naver.com

**Keywords:** *Argopecten irradians*, *Mimachlamys crassicostata*, *Mizuhopecten yessoensis*, *Scaeochlamys farreri*, *Atrina pectinata*, *Swiftopecten swiftii*, multiplex PCR, species identification

## Abstract

Processed bivalve products typically lack diagnostic shell characters, making DNA-based assays essential for reliable species authentication in routine testing. We developed and validated a conventional, COX1-based multiplex PCR for the simultaneous identification of six commercially important food bivalves: *Argopecten irradians*, *Mimachlamys crassicostata*, *Mizuhopecten yessoensis*, *Scaeochlamys farreri*, *Atrina pectinata*, and *Swiftopecten swiftii*. The assay yielded robust, single-band amplification across an annealing window of 54–59 °C and maintained reproducible performance over template inputs from 0.1 to 50 ng per reaction. This work provides a practical marker set for simultaneous identification of six bivalve species in freeze foods or tissues where morphological traits are not preserved.

## 1. Introduction

*Scaeochlamys farreri* and *Mizuhopecten yessoensis* are among the most economically important bivalves farmed in East Asia [1,2]. *S. farreri* and *M. yessoensis* are important farmed scallops in many countries and are of key economic value in the fisheries economy, playing a central role in large-scale scallop aquaculture production (millions of tons) [3,4]. Other scallops such as and *Mimachlamys crassicostata*, as well as and *Atrina pectinata*, are likewise valuable edible species that are widely cultivated or harvested for human consumption [5,6,7,8]. These bivalves provide high-quality protein and hold significant commercial value in domestic and global seafood markets, making them popular among consumers [1,2,5,6,7,8,9]. Studies of commercial scallop products in Europe have shown that scallop mislabeling leads to consumer fraud and poor fisheries management [10]. Furthermore, extensive meta-analyses and regulatory reports indicate that seafood mislabeling often involves substituting lower-value species for higher value products, resulting in significant economic losses and a loss of consumer confidence [10,11,12].

Bivalve products are often distributed as frozen or shell-removed meat, which eliminates morphological characteristics needed for species identification and thus necessitates DNA-based methods for accurate authentication [13]. In the absence of external shell features, molecular diagnostic techniques offer the only reliable means to determine the species of such processed bivalve materials, ensuring proper labeling and prevention of food fraud [13,14,15,16,17].

Multiplex PCR has been adopted internationally (including domestically) as an effective tool to prevent seafood and meat adulteration by enabling simultaneous identification of multiple species in a single test [17,18]. This technique offers high efficiency and cost-effectiveness by amplifying multiple DNA targets at once, providing a rapid and reliable method for species authentication with minimal sample input and no need for specialized equipment [18]. Recent studies have demonstrated that multiplex PCR can sensitively detect the presence of various animal source ingredients in mixed or processed products, thereby safeguarding consumers and enhancing traceability in the food supply chain [19,20,21,22,23,24].

COX1 exhibits relatively low intraspecific variation and high interspecific divergence, maintaining stability within a species while enabling reliable discrimination between closely related species [25]. Furthermore, the conserved flanking regions facilitate robust primer design, and the internal sequences provide sufficient polymorphism to generate species-specific amplicons of different sizes in a single multiplex reaction [26]. Furthermore, COX1 is the standard DNA barcoding region for animals, and extensive reference sequences for various bivalve species are available in public databases [27]. Direct sequencing of the COX1 barcode yields the richest information for species discovery and phylogenetics, but it is poorly suited to routine food authentication workflows [28,29]. Sequencing workflows involve multiple intricate steps and substantial investment of time and money, and they depend on access to specialized equipment as well as considerable bioinformatics infrastructure for subsequent data handling and refinement [29]. As a result, settings that handle large sample volumes under strict time and budget constraints, such as routine quality-control facilities or border inspection checkpoints, are more practically managed with straightforward amplicon-based assays than with comprehensive sequencing approaches [29]. Species-specific PCR is faster, yet it scales poorly because each candidate taxon must be tested in separate reactions [30,31]. By contrast, multiplex PCR can interrogate multiple taxa in a single tube and has been successfully applied to seafood authentication; however, prior work has typically targeted limited panels rather than a comprehensive bivalve set [14,19,21,22,24]. Prior the study, no study has reported a conventional multiplex assay that simultaneously identifies all six major food species considered here, *Argopecten irradians*, *Mimachlamys crassicostata*, *Mizuhopecten yessoensis*, *Scaeochlamys farreri*, *Atrina pectinata*, and *Swiftopecten swiftii*, in one reaction.

The objectives were threefold: (1) to mitigate food fraud by achieving simultaneous, rapid, and definitive identification of six commercially important bivalves, *Argopecten irradians*, *Mimachlamys crassicostata*, *Mizuhopecten yessoensis*, *Scaeochlamys farreri*, *Atrina pectinata*, and *Swiftopecten swiftii*; (2) to establish a COX1-based multiplex PCR assay; and (3) to validate the assay for deployment in food certification programs and regulatory enforcement.

## 2. Materials and Methods

### 2.1. Sample Collection and Genomic DNA Isolation

In January 2021, specimens representing six bivalve species were obtained from seafood retailers in South Korea (Table S1) [32,33,34,35,36,37]. Indeed, the six species examined in this study can be reliably distinguished by their overall shell morphology, number and strength of ribs, coloration and ornamentation, and hinge structure, as described in standard taxonomic references for *A. irradians*, *M. crassicostata*, *M. yessoensis*, *S. farreri*, *A. pectinata*, and *S. swiftii*. All specimens were identified at the time of purchase by a skilled malacologist based on these shell characteristics, in accordance with published identification tables and species descriptions. Adductor muscle was excised from each species, and the samples were immediately frozen at −20 °C and placed in 1.5 mL tubes containing 99.9% ethanol suitable for DNA analysis. Given that bivalve products are typically distributed frozen, we prepared the samples in a frozen state. Genomic DNA was extracted using a freeze–ethanol procedure in which tissues were initially frozen and subsequently immersed in ethanol, then stored at 4 °C for 3 days before DNA isolation. Before extraction, tissues were washed with 3DW water, and genomic DNA was then purified with the HiGene™ Genomic DNA Prep Kit (Biofact, Daejeon, Republic of Korea) according to the manufacturer’s instructions.

### 2.2. Design of Species-Specific Primers for Six Bivalve Species

We obtained the complete sequences of each species from NCBI (EU023915: *Argopecten irradians*; FJ415225: *Mimachlamys crassicostata*; NC_009081: *Mizuhopecten yessoensis*; EU715252: *Scaeochlamys farreri*; KC153059: *Atrina pectinata*; MW646296: *Swiftopecten swiftii*) and extracted the cytochrome oxidase subunit I (COX1) region from six species using Geneious Prime ver. 2025.02 [38]. Species-specific sequences were identified from these regions to design amplifiable bivalve primer sets, and six multiplex PCR primer sets were subsequently developed for amplification.

### 2.3. Species-Specific Multiplex PCR Primer Set Amplification

PCR was performed in 20 μL reactions using the AccuPower^®^ Multiplex PCR Premix (Bioneer Inc., Daejeon, Republic of Korea). Each reaction contained 10 ng of template DNA and all six bivalve specific primer pairs (10 μM each). Thermal cycling consisted of 94 °C for 5 min; 34 cycles of 94 °C for 30 s, 55 °C for 30 s, and 72 °C for 30 s; followed by a final extension at 72 °C for 7 min. PCR amplification products were confirmed on a 1.5% agarose gel.

### 2.4. Determining the Efficient Annealing Temperature of a Multiplex PCR Primer Set

Multiplex PCR was performed as described in Materials and Methods (Section 2.3), except that, to identify the optimal annealing range, the annealing temperature was tested at six settings (54, 55, 56, 58, 59, and 60 °C).

### 2.5. Assessment of Multiplex PCR Performance at Varying DNA Concentrations

Multiplex PCR was performed with the multiplex primer sets using six species genomic DNA templates at four input amounts (50, 10, 1, and 0.1 ng reaction) to confirm amplification. PCR conditions were identical to those in the Materials and Methods Section (Section 2.3). Band intensity measurements for each DNA concentration and species were obtained by digital analysis in ImageJ ver. 1.54s6 [39].

## 3. Results and Discussion

### 3.1. Design of Species-Specific Primers for the Mitochondrial COX1 Region

We designed the forward primers for all six species to carry a species-specific base at the 3′ end, thereby restricting PCR amplification to the intended species. Placing a species-diagnostic base at the 3′ terminus of the forward primer is critical for multiplex specificity, as DNA polymerases poorly extend from 3′-mismatched primers, thereby confining amplification to the target species [40,41,42,43,44,45]. The six species common reverse primers are designed to find the most conserved sequences in all six species as much as possible, and if there are some sequence variations, reverse primers are designed using mixed-base codes (e.g., T and A is W). In multiplex PCR, a single consensus reverse primer combined with species-specific forward primers can reliably discriminate multiple targets; when inter-target variation exists at the reverse-primer binding site, incorporating IUPAC degenerate bases allows comprehensive amplification across variant templates [43,44].

### 3.2. Multiplex PCR Set Amplification

**Figure 1:**
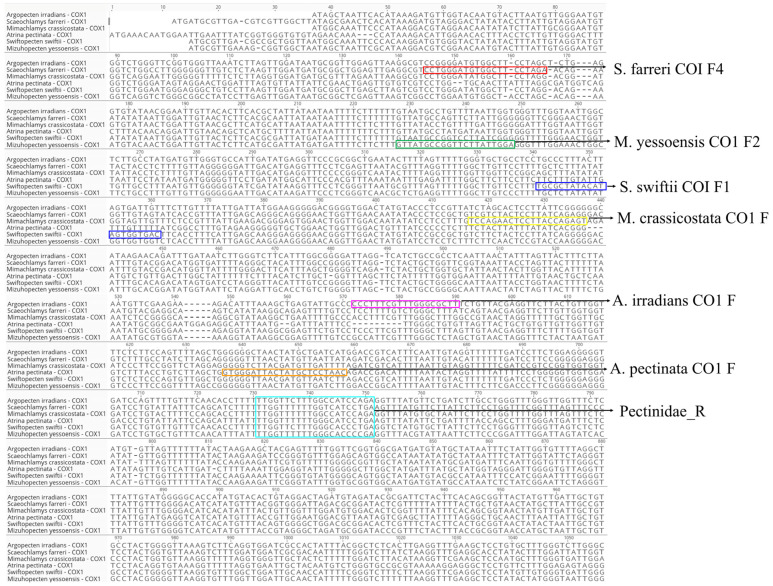
Species-specific multiplex PCR primer design information based on COX1 gene partial sequence alignment for six species. The colored squares represent primer of each species. Colored boxes indicate the species-specific primer annealing sites on the aligned COX1 sequences: red, *S. farreri* COI_F4; green, *M. yessoensis* COI_F2; blue, *S. swiftii* COI_F1; yellow, *M. crassicostata* COI_F; magenta, *A. irradians* COI_F; cyan, *A. pectinata* COI_F. The conserved binding site of the common reverse primer Pectinidae_R is also highlighted. The common reverse primer region of Pectinidae_R is shown as a wide rectangle to indicate that it is the region most conserved among the six species.

**Figure 2:**
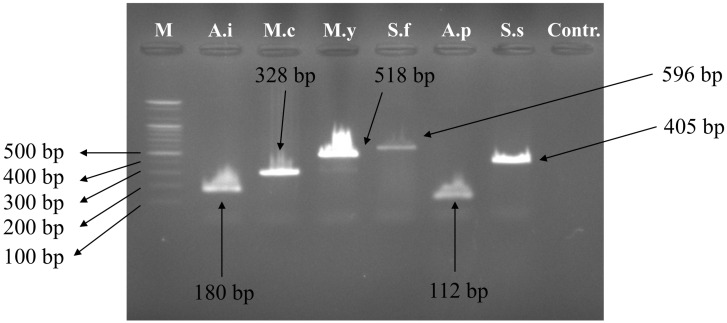
PCR amplification with species-specific multiplex PCR primer sets in six bivalves. M: Represents DNA ladder. Abbreviations: A.i, *Argopecten irradians*; M.c, *Mimachlamys crassicostata*; M.y, *Mizuhopecten yessoensis*; S.f, *Scaeochlamys farreri*; A.p, *Atrina pectinata*; S.s, *Swiftopecten swiftii*, Contr.: Negative control.

Multiple primer sets are provided based on the COX1 sequences of six bivalve species (Table 1). Using specific primer sets for each of the six bivalve species, we obtained bands that were specifically amplified for each species (Figure 1). Each species successfully generated amplicons at the expected amplification site (*Scaeochlamys farreri*: 596 bp, *Mizuhopecten yessoensis*: 518 bp, *Swiftopecten swiftii*: 405 bp, *Mimachlamys crassicostata*: 328 bp, *Argopecten irradians*: 180 bp, *Atrina pectinata*: 112 bp) (Figure 2). In species, discriminating multiplex PCR, assay performance is highly contingent on primer design including the selection of species-unique region, management of amplicon size spacing, and, where necessary, the judicious use of degenerate bases as demonstrated in multiple seafood authentication assays [19,21,22]. Collectively, our findings suggest that a panel of six species-specific markers, distinguished by amplicon length, can provide clear discrimination among commonly consumed taxa and may be applicable to foods or tissue samples in which diagnostic morphological characters are not preserved.

### 3.3. Multiplex PCR Amplification Efficiency Across Annealing Temperatures

**Figure 3:**
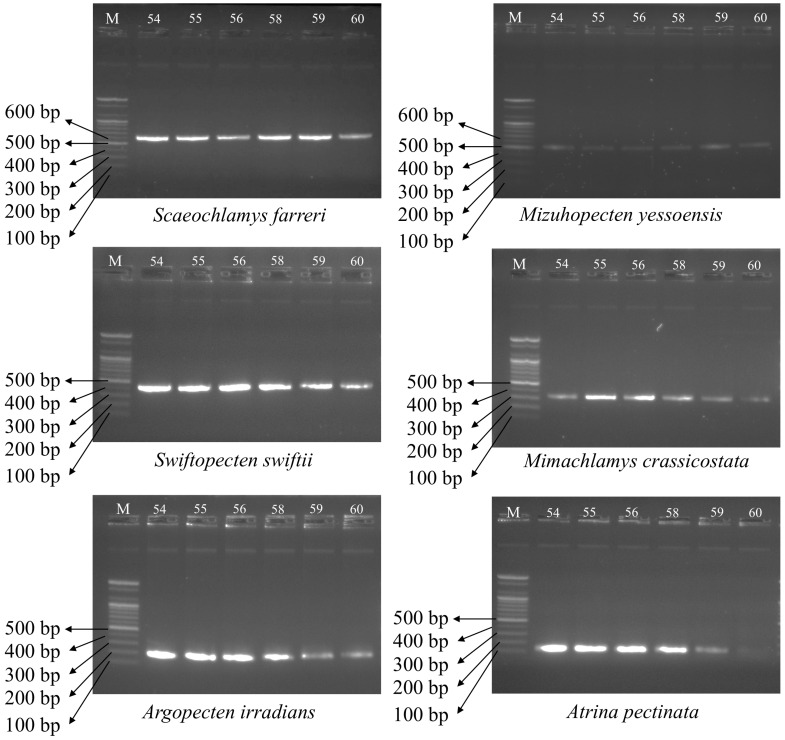
Multiplex PCR amplification profiles obtained with the primer set for six bivalve species. M: Represents DNA ladder. The numbers (54–60 °C) in the electrophoresis image are the annealing temperatures.

The six species multiplex PCR sets were tested over an annealing temperature range of 54–60 °C, with efficient amplification achieved from 54 to 59 °C (Figure 3). Prior work has likewise established workable annealing conditions for multiplex PCR [23]. In this context, optimizing temperature is important, as the setting that promotes single-band, species-specific amplification with high yield underpins robust identification and helps prevent mislabeling and adulteration in seafood products.

### 3.4. Sensitivity of Multiplex PCR Amplification Across DNA Concentrations

**Figure 4:**
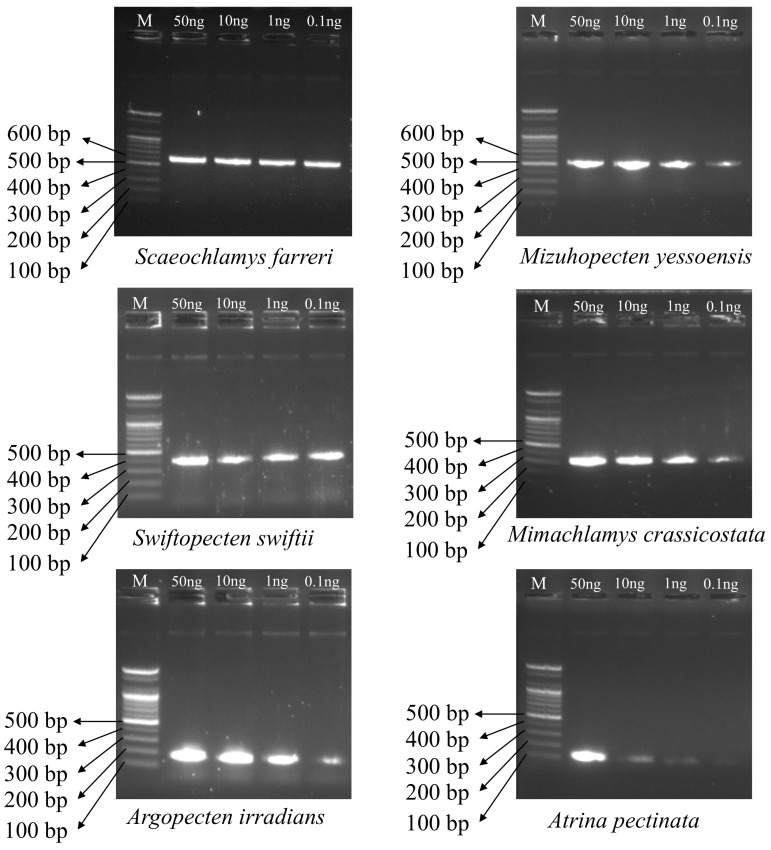
Multiplex PCR amplification of six bivalve species across template DNA concentrations (0.1–50 ng); band patterns analyzed for each species.

We established a COX1-based multiplex PCR assay capable of identifying six bivalve species and obtained consistent amplification over a template DNA range of 0.1–50 ng (Figure 4). The analytical sensitivity aligns with prior reports for food mitochondrial marker multiplexes, which typically reach a detection limit of ~0.1 ng [20,23,45]. Notably, despite the limited number of published applications to domestic and international food products for these six taxa, the panel yielded stable amplification at the 0.1 ng threshold. These findings indicate that a COX1 based multiplex can support rapid, reliable identification in processed seafood matrices when ≥0.1 ng of DNA is available or when cross-contamination is suspected. We did not assess performance following salting or heat treatment, so additional validation under those conditions will be required. Within these bounds, the approach appears well suited to verification needs in food hygiene and origin-labeling contexts and is applicable to small inputs and low DNA concentration extracts, providing a practical marker set for simultaneous identification of the six bivalve species.

## 4. Conclusions

We developed a COX1-based conventional multiplex PCR that simultaneously identifies six commercially important bivalves (*Argopecten irradians*, *Mimachlamys crassicostata*, *Mizuhopecten yessoensis*, *Scaeochlamys farreri*, *Atrina pectinata*, and *Swiftopecten swiftii*) in a single reaction. The assay produces clearly size resolved amplicons, operates across a broad annealing range (54–59 °C), and detects as little as 0.1 ng of DNA. A primer design combining species-diagnostic 3′ end forward primers with a degenerate consensus reverse primer yielded target-specific bands suitable for routine gel electrophoresis. These features enable rapid, low-cost screening without specialized instrumentation, supporting practical use in quality-control laboratories and regulatory agencies to mitigate seafood mislabeling and adulteration.

## Figures and Tables

**Table 1:** Multiplex PCR primer sets designed from the COX1 region of six bivalve species.

Primer Name	Scientific Name	Sequence (5′–3′)	Primer Direction	Expected Amplification Product Size (bp)
*S. farreri* COI F4	*Scaeochlamys farreri*	CCTGGGATGTGGCTTCCTAGA	Forward	596
*M. yessoensis* CO1 F2	*Mizuhopecten yessoensis*	GTTATGCCGGTTCTTATTGGA	Forward	518
*S. swiftii* COI F1	*Swiftopecten swiftii*	TGCGCTATACATAGTGGTGAC	Forward	405
*M. crassicostata* CO1 F	*Mimachlamys crassicostata*	TCCAGAACTCCTTACCAGAGT	Forward	328
*A. irradians* CO1 F	*Argopecten irradians*	CCCTTTCGTTTGGGCGCTT	Forward	180
*A. pectinata* CO1 F	*Atrina pectinata*	GTGGGATTACTATGCTCCTAAC	Forward	112
Pectinidae_R	-	TCDGGRTGVCCAAARAAYCAA	Reverse	-

## Data Availability

The data presented in this study are available on request from the corresponding author.

## Supplementary Materials

The following supporting information can be downloaded at https://www.mdpi.com/article/10.3390/foods14223881/s1. Figure S1: Quantitative band measurements for six bivalve species. Table S1: Sampling sites of six bivalve species in the study.
