# Supplementary material for: Development of a Multiplex PCR Assay for Simultaneous Identification of Six Commercially Important Bivalves
Source: Foods. 2025 Nov 13;14(22):3881. doi: 10.3390/foods14223881 (PMC12650985; doi:10.3390/foods14223881)
Supplement: Supplementary file 1 [file foods-14-03881-s001.zip › foods-3969151-supplementary.pdf]

Table S1. Sampling sites of six bivalve species in the study

Scientific name	Location	Location	Sample date	Morphological reference
Argopecten irradians	Goseong	38°23'13″ N, 128°27'42" E	2021.01	Marelli & Arnold, 2001[1]
Mimachlamys crassicostata	Tongyeong	34°50'24″ N, 128°25'20" E	2021.01	Su et al., 2023[2]
Mizuhopecten yessoensis	Goseong	38°23'13″ N, 128°27'42" E	2021.01	Pravdoukhina & Kodolova, 2011[3]
Scaeochlamys farreri	Tongyeong	34°50'24″ N, 128°25'20" E	2021.01	Lutaenko & Noseworthy, 2019[4]
Atrina pectinata	Boryeong	36°21'04″ N, 126°36'02" E	2021.01	Xue et al. 2021[5]
Swiftopecten swiftii	Goseong	38°23'13″ N, 128°27'42" E	2021.01	Yoshimura et al. 2019[6]

Reference:

[1]Marelli, D. C., & Arnold, W. S. (2001). Shell morphologies of bay scallops, *Argopecten irradians*, from extant and prehistoric populations from the Florida Gulf Coast: Implications for the biology of past and present metapopulations. *Journal of Archaeological Science*, 28(6), 577–586.;

[2]Su, J., Liu, B., & Zhu, C. (2023). Morphological trends in three different populations of the noble scallop *Mimachlamys crassicostata* (GB Sowerby II, 1842) along the South China Sea Coast and their relationship to environment factors. *Indian J. Fish*, *70*(1), 10-17.;

[3]Pravdukhina, O. Y., & Kodolova, O. P. (2010). Temporal dynamics of genetic diversity of Japanese scallop *Mizuhopecten yessoensis* (Jay, 1856). *Biology Bulletin*, *37*(4), 363-373.;

[4]Lutaenko, K. A., & Noseworthy, R. G. (2019). Contribution to the knowledge of the marine bivalve mollusk fauna of Gangwon Province, Korea. *Journal of Asia-Pacific Biodiversity*, *12*(1), 14-44.;

[5]Xue, D. X., Wang, H. Y., & Zhang, T. (2021). Phylogeography and taxonomic revision of the pen shell *Atrina pectinata* species complex in the south China sea. *Frontiers in Marine Science*, *8*, 753553.;

[6]Yoshimura, T., Shirai, K., Murakami-Sugihara, N., Sakai, S., & Sasaki, T. (2019). Sexual dimorphism in shell growth of the oviparous boreal scallop *Swiftopecten swiftii* (Bivalvia: Pectinidae). *Journal of Molluscan Studies*, *85*(2), 253-261.

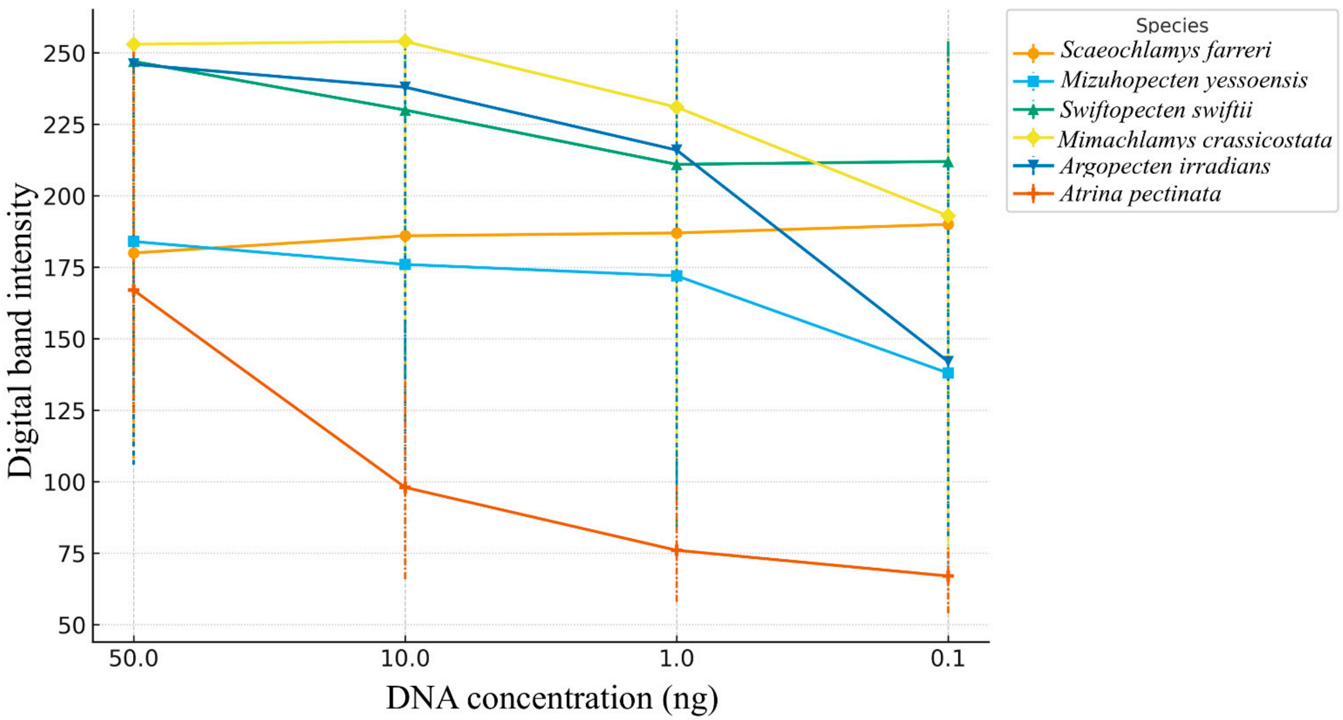

Figure S1. Quantitative band measurements for six bivalve species. Line colors denote the species-specific digital quantification values. The error bars show the minimum and maximum ranges, while the central line marks the average.
